# CTCF-anchored chromatin loop dynamics during human meiosis

**DOI:** 10.1186/s12915-025-02181-3

**Published:** 2025-03-20

**Authors:** Vera B. Kaiser, Colin A. Semple

**Affiliations:** MRC Human Genetics Unit, Institute of Genetics and Cancer, University of Edinburgh, Western General Hospital, Crewe Road, Edinburgh, EH4 2XU UK

**Keywords:** Chromatin loops, Recombination, Single cell, RNA-seq, ATAC-seq, Machine learning

## Abstract

**Background:**

During meiosis, the mammalian genome is organised within chromatin loops, which facilitate synapsis, crossing over and chromosome segregation, setting the stage for recombination events and the generation of genetic diversity. Chromatin looping is thought to play a major role in the establishment of cross overs during prophase I of meiosis, in diploid early primary spermatocytes. However, chromatin conformation dynamics during human meiosis are difficult to study experimentally, due to the transience of each cell division and the difficulty of obtaining stage-resolved cell populations. Here, we employed a machine learning framework trained on single cell ATAC-seq and RNA-seq data to predict CTCF-anchored looping during spermatogenesis, including cell types at different stages of meiosis.

**Results:**

We find dramatic changes in genome-wide looping patterns throughout meiosis: compared to pre-and-post meiotic germline cell types, loops in meiotic early primary spermatocytes are more abundant, more variable between individual cells, and more evenly spread throughout the genome. In preparation for the first meiotic division, loops also include longer stretches of DNA, encompassing more than half of the total genome. These loop structures then influence the rate of recombination initiation and resolution as cross overs. In contrast, in later mature sperm stages, we find evidence of genome compaction, with loops being confined to the telomeric ends of the chromosomes.

**Conclusion:**

Overall, we find that chromatin loops do not orchestrate the gene expression dynamics seen during spermatogenesis, but loops do play important roles in recombination, influencing the positions of DNA breakage and cross over events.

**Supplementary Information:**

The online version contains supplementary material available at 10.1186/s12915-025-02181-3.

## Background

Recombination, the generation of novel combinations of alleles, is a critical process that affects an individual organism’s phenotype, population-level amounts of genetic diversity and the response to selection [1, 2]. In mammals, homologous recombination occurs during meiosis, the cytological process that gives rise to gametes which provide all genetic information for the next generation [3, 4]. Prophase I of meiosis is a lengthy and complex process and is subdivided into five different stages (leptotene, zygotene, pachytene, diplotene and diakinesis), which, together, last for several days in human males. Prophase I takes place in primary spermatocytes, where programmed DNA double-strand breaks (DSBs) are introduced, homologous chromosomes pair at the synaptonemal complex and exchange genetic material [5]. When DSBs occur during pachytene, overhanging single-stranded DNA (ssDNA) is bound by DMC1 near the breakage site [6]. A fraction of such DMC1-bound ssDNA sites result in strand exchange and crossovers [7], but the underlying process by which sites are chosen remains unclear [8]. At the population level, cross-over events are concentrated in recombination hotspots (HSs), which are typically 1–2 kb in size [9–11], suggesting strong biases. Previously [12], we showed that HSs may be a by-product of particular chromatin environments, defined by patterns of chromatin looping, but chromatin data from human meiotic cells were not available at the time. However, the recent emergence of scATAC-seq data from human testicular samples [13] has provided new opportunities to study the dependencies between the process of recombination and chromatin structure in humans.

Chromatin looping is a fundamental level of organisation in all eukaryotic cells, including germline cells. In somatic cells, chromatin topology can be studied by methods such as Hi-C [14], ChIA-PET [15] and related methods. In the current widely accepted model, the cohesin complex catalyses the formation of chromatin loops at the scale of hundreds of kilobases (kb), and loops are anchored at convergently oriented CTCF-binding sites, which prevent the cohesin molecules from releasing the DNA [16]. During interphase, chromatin loops appear to regulate gene expression [17]; during meiosis, they may play a crucial role in a different context—the pairing of the homologous chromosomes at the synaptonemal complex. In mice, stage-resolved Hi-C analysis of spermatogenesis has been performed via the experimental synchronisation of germ cells [18, 19]; this has demonstrated that, while larger chromatin domains are lost in prophase I [20, 21], CTCF retains its insulator function [22] and may be recruited to the synaptonemal complex (SC) via co-localisation with RAD21/SMC1/SMC3 [23–25], although the details of interaction between CTCF and the SC remain to be demonstrated. Chromatin loops are known to persist throughout meiosis [5], and their anchor points coincide with CTCF binding [26]; however, the size of loops throughout prophase I is contentious [27] as loop sizes of 0.8–2 Mb have been measured by Hi-C experiments in mice [18, 21, 28], but computational analyses [8] have revealed shorter loops in A compartments—similar to typical interphase loops, which are of the order of 200 kb [29]. Overall, the topological organisation of the human genome during meiosis remains elusive. In particular, the positions of the genome that form loops during human meiosis are currently unknown, as is the level of variation in looping patterns between individual cells. In addition, the relationship between the chromatin structure of meiotic DNA and features of recombination [8] is largely unexplored.

Computational methods that employ machine learning strategies to predict chromatin looping [30–33] are a timely substitute when experimental data are hard to come by. Machine learning models can be trained on experimental data, such as CTCF-binding, gene expression and other epigenetic marks in combination with a set of “true” loops derived from Hi-C or ChiA-Pet datasets. Such models can predict chromatin looping with high accuracy—with AUC values typically > 95%—suggesting that computational approaches can sometimes outperform experimental data at predicting chromatin architecture, especially when sequencing depth is a limiting factor [31]. However, in order to study chromatin looping during spermatogenesis, there is need to specifically study cells that are in the correct stage of meiosis, e.g. cells which undergo DSBs when studying the impact of looping on the process of recombination. One way to obtain sub-populations of cells is by single cell sequencing and selecting cells based on marker gene expression.

Here, we use machine learning methods to study a fundamental level of chromatin organisation during human meiosis: CTCF-anchored loops. We predict chromatin folding during all relevant cell stages and study the impact of chromatin loops on gene expression, DSB initiation and homologous recombination patterns across the genome.

## Results

### Dynamics of CTCF mediated chromatin loops during spermatogenesis

**Fig. 1:**
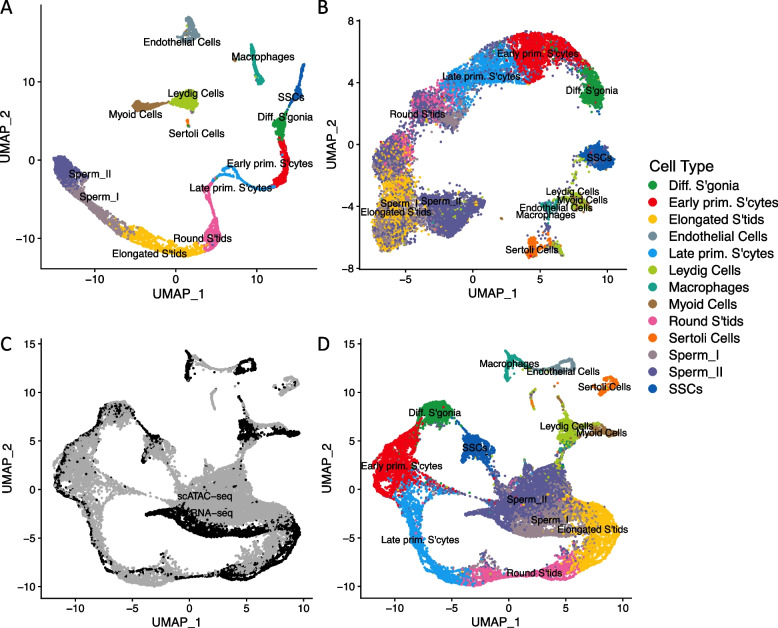
Single cell expression and chromatin accessibility patterns during human spermatogenesis. Dimension reduction (UMAP) plots of scRNA-seq (**A**) and scATAC-seq (**B**) dataset; cell state annotations are derived from marker gene expression in (**A**) [34]. The same data are projected onto a single plot, with the population of origin indicated in (**C**) and cell annotations shown in (**D**). In (**C**), scATAC-seq data are shown in grey and scRNA-seq data in black

We interrogated a recently published testicular scATAC-seq dataset [13] to identify human meiotic cells. The cellranger-atac pipeline detected a combined total of 20,296 cells in the dataset (10,509 cells in SRR21861961, 7086 in SRR21861962 and 2701 in SRR21861963). Next, cell states were inferred using matched single cell RNA-seq data [34]. Using marker gene expression, chromatin accessibility and the computation of “transfer anchors”, scRNA-seq profiles were used to project cell identities from the annotated scRNA data onto the scATAC-seq dataset (Fig. 1A) [35]. As in Guo et al. [34], we classified cells into eight cell types of germ cell development: spermatogonial stem cells (SSCs), differentiating spermatogonia, early primary spermatocytes, late primary spermatocytes, round spermatids, elongated spermatids, sperm I and sperm II. For each cell type, we identified between ~ 700 and 4 thousand cells, including 2008 early primary spermatocytes, i.e. diploid cells preparing for the first meiotic division. A pseudo time-course of meiosis is shown in the batch corrected UMAP plot (Fig. 1B) [36], displaying the transitions in chromatin accessibility across cells from SSCs to mature sperm. For illustrative purposes, we also show the scRNA-seq and scATAC-seq data projected onto the same plot (Fig. 1C and D), illustrating how cell identities were transferred from the gene expression dataset onto the chromatin accessibility dataset.

**Fig. 2:**
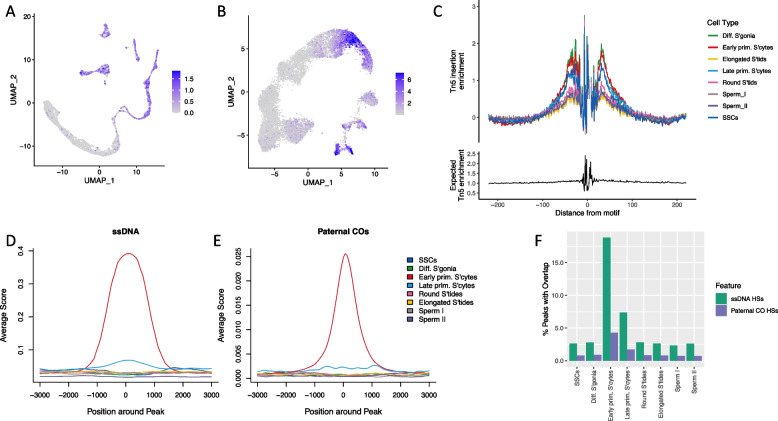
Dynamics of CTCF activity and recombination during meiosis. CTCF activity is visualised in the scRNA-seq (**A**) and scATAC-seq data (**B**), using the same embeddings as in Fig. 1. **C** CTCF binding-site footprinting profiles derived from the scATAC-seq data, using Signac [35]. Cell-type-specific peaks were profiled for ssDNA overlap (**D**) as well as paternal cross-over activity (**E**), using the genomation package in R [43]. **F** The 80% reciprocal overlap percentage for all scATAC-seq peaks in a given cell type versus ssDNA HSs (green) and paternal CO hotspots (purple)

In order to infer chromatin loops accurately in any given cell type, it is crucial to assess CTCF binding activity first. CTCF is known to be widely expressed in humans [37, 38], including in spermatogenesis [39]. Post-meiotic cells have a highly compacted genome, with histones being replaced by protamines [40] and transcription largely, but not completely, silenced after the histone-to-protamine transition [41]. However, CTCF binding is known to be active in the regions of the haploid sperm genome that escape this transition [42]. We predicted bound CTCF sites in all cell types based upon established footprinting analysis within ATAC-seq peaks (Methods). The overall trajectories of CTCF activity were similar in the scRNA-seq and scATAC-seq datasets studied here—as expected if CTCF gene expression results in increased binding. Activity was found to be highest in SSCs and before the first meiotic division, including in early primary spermatocytes; it was then dramatically reduced in round spermatids and beyond, in accordance with a general compaction of the genome (Fig. 2A, B). However, CTCF activity could be measured across all cell types, including in mature sperm. In addition, binding site footprinting plots [35] show a shoulder around CTCF-motifs in all cell types, reflecting a higher-than-expected transposase insertion frequency around the CTCF motif also in post-meiotic cells and thus indicating protein binding at the CTCF motif sites, albeit at reduced intensity (Fig. 2C).

**Fig. 3:**
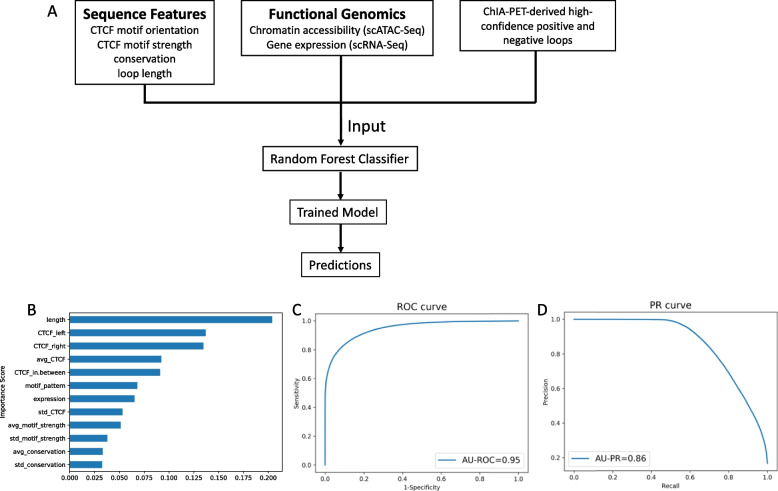
Accurate prediction of chromatin loops using single cell data. A machine learning model was trained on aggregated scRNA-seq and scATAC-seq data in GM12878, using ChIA-PET and Hi-C data as ground truths. **A** Overview of the pipeline, which yields a random forest classifier and can be used to make predictions on a new dataset. **B** Feature importance scores = mean decrease impurity in the training process; “avg” and “std” represent the mean and standard deviation of the signal intensity on both anchors; “left” and “right” represent flanking features and “in-between” is the signal intensity within a loop. **C** Receiver operator characteristic (ROC) curve and **D** precision recall (PR) curve, using five iterations of tenfold cross validation

Having confirmed CTCF binding activity, we employed a machine learning framework to study the changing chromatin conformation landscape throughout spermatogenesis. We used an established random forest modelling approach for bulk data that allows for de novo prediction of chromatin loops [31] and adapted this framework to single cell datasets; we trained the model on aggregated pseudo-bulk scATAC-seq and scRNA-seq data derived from the GM12878 lymphoblastoid cell line (Methods). Using tenfold cross validation, the model performed well on the GM12878 single cell test data and achieved an area under ROC curve value of 0.95, based on ground truth ChIA-PET and Hi-C datasets of chromatin loops. Loop length and CTCF signal intensities at loop anchors achieved the highest importance scores (Fig. 3).

The model was further validated in an independent single cell dataset from the K562 cell line; using a 99% reciprocal overlap criterion, almost all of the experimentally derived ChIA-PET loops (99%) were predicted by our model (Additional file 1: Table S1). Additional loops that were predicted by the machine learning model but absent in the ChIA-PET data seem to be due to a lack of sequencing depth in the latter, since lowering the minimum PET (paired-end tag) support required for a ChiA-PET loop to be included recovers an increasing number of predicted loops (Additional file 1: Table S1), with a maximum of 59% of predicted loops supported experimentally when ChIA-PET loops are supported by at least 3 PETs.

We further tested the robustness of our model by comparing it to (a) a model trained on chromosomal cross-fold validation schemes [44] and (b) a model that only used CTCF features for model training (see Methods). Both alternative models resulted in comparable AU-ROC and AU-PR scores as well as a similar estimation of loop numbers in the different spermatogenesis cell types (Additional file 2: Fig. S1).

### Distinct chromatin conformations emerge in preparation for meiosis

**Fig. 4:**
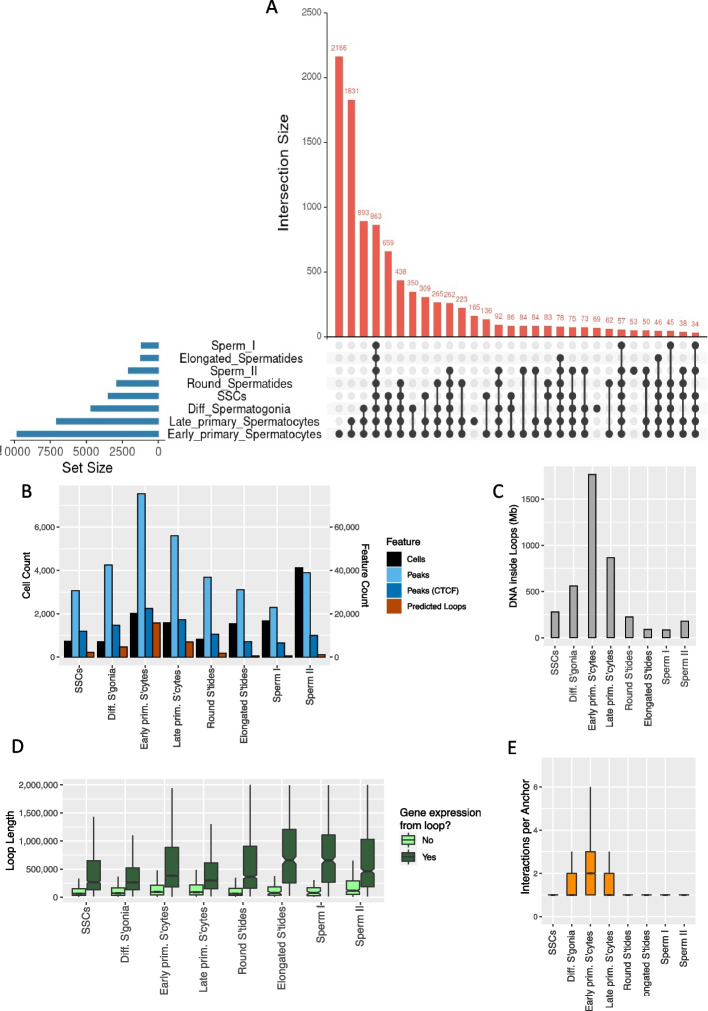
Characteristics of predicted loops in germ cell development. **A** Upset plot of the number of predicted loops in each cell state and their mutual overlap. **B** The total number of cells in the scATAC-seq dataset assigned to a given cell state (black bars, left Y axis). The total number of scATAC-seq peaks called in each of the cell states (light blue), the number of peaks containing CTCF footprints (dark blue) and the number of predicted loops (red) are shown on the right hand Y axis. **C** The total amount of unique DNA covered by loops annotated in each of the germ cell populations. **D** The distribution of loop lengths for loops that contain genes which are expressed, compared to loops that do not contain expressed genes. **E** The number of predicted interacting anchors per CTCF-anchor. Boxplots in **D** and **E** indicate the median (central bar), the 25th and 75.th percentiles (boxes), and 95% percentiles (whiskers)

We used the trained model to predict chromatin loops in each of the eight cell types separately, using cell-type specific input features of CTCF-binding, chromatin accessibility and gene expression. Among cell types within the spermatogenesis dataset, the number of predicted loops ranged from 520 in sperm I, to 15,693 in early primary spermatocytes (Fig. 4 and Additional file 3: “Predicted Loops”). However, the number of predicted loops was not a simple consequence of cell numbers in a given cell type. For example, mature sperm (sperm II) had the highest cell count, but, consistent with a compaction of the haploid genome, we found only a moderate number of accessible peaks and a strongly reduced number of predicted loops, relative to other cell types (Fig. 4). Conversely, in the same cells, regions surrounding genes known to be specifically active during the later stages of spermatogenesis showed high signals of accessibility. Examples of such genes are TNP1 and PRM2, both of which are involved in chromatin remodelling of the haploid genome and show accessibility almost exclusively in the haploid phase (Additional file 2: Fig. S2).

Pairing and synapsis of homologous chromosomes takes place at the early primary spermatocyte stage, and various interconnected aspects of predicted chromatin structure reach notable maxima, including chromatin accessibility (numbers of scATAC-seq peaks), CTCF binding and numbers of chromatin loops (Fig. 4A, B, C). Compared to earlier and later time points, early primary spermatocytes also stood out as having more complex and variable loop structures, with a higher number of interactions measured for a given loop anchor site (Fig. 4E). Chromatin loops in early primary spermatocytes contained by far the largest amount of unique DNA (1766 Mb; Fig. 4C), with a median distance of 390 kb between non-overlapping loops. Overall, loops increase in size in the transition from SSCs to early primary spermatocytes—to around half a megabase, on average—and then decrease again in late primary spermatocytes, with the presence of expressed genes being associated with longer loop lengths (Fig. 4D). Note that this association between loop length and expression is not unique to the spermatogenesis dataset, but was also found in the GM12878 training set, with median loop lengths of 87 kb and 476 kb for loops with zero and non-zero expression levels, respectively; Wilcoxon test: *W* = 4,817,254,898, *p* < 10^^−16^.

### A post-meiotic shift of loops to the telomeres

**Fig. 5:**
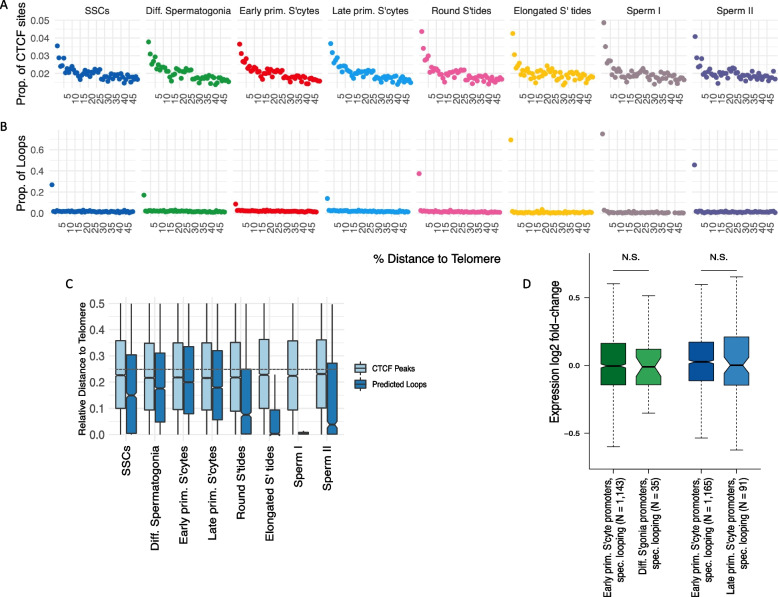
The dynamics of chromatin loop location during spermatogenesis and the impact of looping on gene expression change. In (**A**) and (**B**), chromosomes were divided into 100 even-sized bins; the X axis shows the distance of a given bin to the nearest telomere, relative to chromosome length. On the Y axes, we plot, on a per-cell type basis and across all chromosomes: (**A**) the proportion of footprinted CTCF sites in a given bin, relative to the total number of footprinted CTCF sites; (**B**) the proportion of predicted loops in a given bin, relative to the total number predicted loops. **C** The distance to the nearest telomere, relative to chromosome length, is shown for CTCF motifs (light blue) and predicted loops (dark blue). An average relative distance of 0.25 is expected if elements are evenly distributed with respect to telomeric distance, indicated by the dashed line. **D** Looping does not result in gene activation in early primary spermatocytes. Y axis: log2 fold-change of gene expression, with log2 fold-values > 0 indicating higher expression in early primary spermatocytes. The number (*N*) of promoters located in cell-type specific loops is indicated in each comparison, and the Wilcoxon signed-rank test was used to compare the median log2 fold-change between categories. Green boxplots: early primary spermatocytes versus differentiating spermatogonia; promoters are either in early primary spermatocyte-specific loops (left) or in differentiating spermatogonia-specific loops (right). Blue boxplots: early versus late primary spermatocytes; promoters are either in early (left) or late (right) primary spermatocyte-specific loops

The chromosomal distribution of CTCF sites and loops differed substantially between cell types: active (i.e. scATAC-seq footprinted) CTCF sites were somewhat biased towards the telomeres at all stages of meiosis, with about 4–5% of CTCF sites falling within the 1% outermost ends of the chromosomes (Fig. 5A). However, in the haploid phase, this bias in the distribution of CTCF is dwarfed by the difference in predicted loop density near the telomeres: in sperm I, a total of 75% of loops (390 out 520) are predicted to fall into the 1% outermost bins of sequence (Fig. 5B). These loops do not originate post-meiotically but are already present in SSCs (Fig. 4A), suggesting that, in the haploid phase, loops are lost along the length of the chromosomes, rather than specifically gained near the telomeres, resulting in an enrichment of loops near the chromosomal ends. In stark contrast, the most uniform distribution of loops along the chromosomes is found during prophase I of meiosis, in early primary spermatocytes (Fig. 5B, C).

Overall, these results suggest that chromatin conformation acquires distinct features as CTCF-anchored loops become more abundant in the transition from SSCs to meiotic cells. Loops become more variable between individual cells, reflected by a higher number of interactions per CTCF anchor, increase in size and are more evenly spread across the chromosomes—until they shrink in absolute numbers and are largely confined to the chromosomal ends in haploid cells.

### Chromatin looping does not drive gene expression changes during spermatogenesis

A substantial literature on chromatin loops and related structures, such as TADs (e.g. [45–48]), has suggested these structures have roles in gene regulation, coordinating expression changes in functionally related genes by facilitating enhancer-promoter interactions [49–51]. We tested this idea by contrasting the gene expression change associated with loops that specifically emerged in the transition “differentiating spermatogonia—> early primary spermatocytes” and “early primary spermatocytes—> late primary spermatocytes”. Further, we only considered genes whose promoters were involved in chromatin loops exclusively in early primary spermatocytes or exclusively in the preceding or subsequent cell type (see upset plot in Fig. 4A). If chromatin looping led to promoter activation, the log2-fold ratio of gene expression would be, on average, larger for genes in early spermatocyte-specific loops. However, this was not the case (Fig. 5D), i.e. loops which emerged specifically in prophase I were not associated with the activation of promoters that reside inside these loops. Similarly, the overlap between a promoter and an emerging loop anchor point (plus minus 1 kb) was not associated with a change in gene expression from differentiating spermatogonia to early primary spermatocytes or from early to late primary spermatocytes (Wilcoxon tests: *W* = 699, N.S., and *W* = 231, N.S.).

We further studied the relationship between gene function and chromatin looping by comparing—on a per cell-type level—the gene ontology (GO) enrichments of genes with promoters inside or outside of loops, respectively, ranked by gene expression level [52]. GO categories of highly expressed genes turned out to be very similar between the two categories of genes (Additional file 1: Table S2). For example, in sperm I, the top ten enriched GO categories are identical between genes whose promoters are involved in looping versus genes with promoters outside loops; in both cases, gene function of highly expressed genes mainly relates to mRNA processing and translation, reflecting the silencing of most transcription in sperm and a shift towards post-transcriptional processing of existing transcripts [53]. Similar results (no difference in GO categories) obtained for the seven other spermatogenesis cell types (Additional file 1: Table S2) and indicates that loops do not contain genes that are functionally different from non-loop genes during meiosis. Another possibility, which could explain changes in looping, is that chromatin conformation dynamics are largely mechanistically associated with aspects of meiosis itself, such as recombination, and we explored this option further.

### Meiotic chromatin accessibility and chromatin looping are major determinants of global recombination patterns

**Fig. 6:**
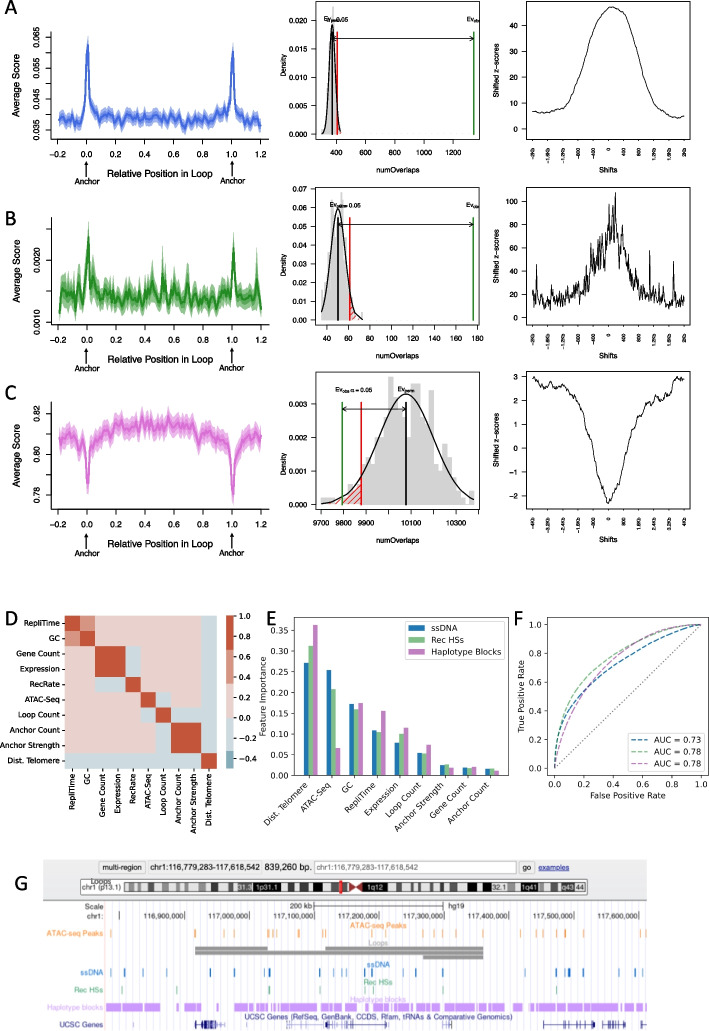
Chromatin looping is associated with DNA breakage and recombination. **A** ssDNA hotspots, (**B**) paternal crossing over events and (**C**) East Asian ancestry (EAS) haplotype blocks were intersected with chromatin loops in early primary spermatocytes (from EAS sample donors). In each row, the first plots show average enrichment scores across all loops (plus/minus 20% of loop length), including, as shaded bands, the standard error of the mean and the 95% confidence interval for the mean. The second plot in each row is a probability density plot based on 1000 circular permutations; the observed number of overlaps between loop anchors and (**A**) ssDNA peaks, (**B**) paternal crossing over events and (**C**) EAS haplotype blocks is indicated by the green vertical lines; in each case the expected distribution of overlap is shown as a grey histogram. The threshold for statistical significance is indicated by the red vertical lines. The last plot in each row shows the distribution of shifted *Z*-scores, i.e. the variation in circular permutation *Z*-scores if loop anchors and ssDNA peaks, paternal crossing over events or EAS haplotype blocks, respectively, were shifted with respect to each other. A distinct peak (or trough) in the local *Z*-scores indicates that overlaps result from specific local patterns. **D** Spearman’s correlation coefficient between genomic features, calculated genome-wide in 1 kb genomic bins. **E** Feature importance plot for the random forest classifier models of ssDNA hotspots (blue), recombination hotspots (green) and EAS haplotype blocks (purple). **F** Receiver operating characteristic (ROC) curve of all three models. **G** Illustrative UCSC genome browser screen shot of the distribution and size of genomic elements. Shown are ATAC-seq peaks in early primary spermatocytes, predicted loops in early primary spermatocytes, ssDNA hotspots, paternal recombination hotspots, EAS haplotype blocks and UCSC gene models

Compared to pre- or post-meiotic germline cell types, ATAC-seq peaks in early primary spermatocytes were most strongly associated with measures of recombination, including ssDNA hotspots [54] as well as paternal cross-over events [55] (Fig. 2D, E and F). A total of 21.6% of accessible sites (16,244/75,351) in early primary spermatocytes overlapped DMC1-bound ssDNA peaks; genome-wide, the overlap represents a more than fivefold enrichment compared to random expectation (*p* = 0.001 with *N* = 1000 circular permutations) and shows that double-strand breaks during prophase I of meiosis are often contained within accessible sites of cells that prepare to undergo chromosome pairing [56, 57]. In addition, using the MEME de novo search algorithm [58], we retrieved an experimentally derived PRDM9 binding motif as the most significant motif from these DMC1-bound/accessible chromatin sites (*E* value = 3.2e − 11; Additional file 2: Fig. S3). Next, we investigated how chromatin loops in early primary spermatocytes relate to recombination events. Dividing loops into even-sized bins and aggregating signals across all loops, we find that ssDNA hotspots and paternal cross-over events are enriched at loop anchors, whereas haplotype blocks often end at loop anchors (Fig. 6A, B, C) [59]. In an independent analysis and using circular permutations, we quantify the genome-wide enrichment of ssDNA hotspots at loop anchors as 3.6-fold, and the enrichment of paternal cross-over locations as 3.5-fold (Table 1). Conversely, haplotype blocks are depleted at loop anchors relative to genome-wide expectations (Table 1) though loops themselves tend to overlap many haplotype blocks (Fig. 6G), i.e. anchors stand out in their genomic context as disrupting haplotype blocks, indicative of historical recombination events at loop anchor sites.

**Table 1:** Circular permutation results. Statistics of overlap between looping and recombination features. The ratio of observed over expected overlaps is shown in column 5, and associated *p* values and permutation *Z*-scores in columns 6 and 7. All results are based on *N* = 1000 permutations

Feature A	Feature B	Overlaps (Obs.)	Overlaps (Exp.)	Ratio	*p*		*Z*
Loop anchors	ssDNA	1340	373.5	3.59	0.0010	46.67	
Loop anchors	COs	176	50.5	3.49	0.0010	18.72	
Loop anchors	Haplotype blocks	9794	10,078.5	0.97	0.0130	− 2.34	
Loops	ssDNA	8159	6896	1.18	0.0010	12.42	
Loops	COs	8984	7839	1.15	0.0010	10.21	
Loops	Haplotype blocks	11,249	10,517	1.07	0.0010	6.42	

Although chromatin conformation appears to play a role in recombination, a variety of other features are known to be important in the broad genome-wide patterns of recombination. To investigate the impact of chromatin folding on recombination while controlling for other intercorrelated factors (Fig. 6D), we employed a set of random forest models to predict genome-wide patterns of recombination in relation to genomic features such as GC content, chromatin accessibility, meiotic replication timing, gene expression levels, chromosomal positioning, as well as variables associated with chromatin loops in prophase I. We modelled, as categorical outcome variables, the presence of (a) ssDNA and (b) paternal recombination hotspots in 1 kb windows along the genome (based on the data of Pratto et al. [54] and Halldorsson et al. [55]), as well as (c) the overlap with EAS haplotype blocks [59]. In all three models, the random forest classifier achieved moderate ROC-AUC scores, and telomeric distance was the most important predictor (Fig. 6E, F); this may reflect the fact that paternal cross-over events are biased towards the tip of the chromosomes [60] and, similarly, Subramanian [61] observed an increase in DSB formation in telomere-proximal regions in yeast. Chromatin accessibility ranked highly in the models of ssDNA and recombination hotspots, consistent with the observation that PRDM9 creates nucleosome-depleted regions [56, 57]; however, previous studies have lacked accessibility data from meiotic human cells. Local GC content and meiotic replication timing also ranked highly across models, and both features are known to correlate with levels of recombination [62, 63]. With respect to looping features, the overlap of a genomic window with the *internal* section of a loop is, in all three models, more important than the number of loops emerging from a given 1 kb bin or the predicted anchor strength in a genomic window (Fig. 6). Thus, while the presence of anchors and loops both contain predictive information on recombination, overall, the internal sequence of chromatin loops is more strongly associated with recombination features than the anchor sites that form the boundaries to loops.

It is an open question why some ssDNA sites are selected as sites of crossing-overs, whereas others are repaired without this type of chromosomal exchange [64]. Hence, we partitioned ssDNA HSs based on their overlap with paternal recombination HSs and investigated how chromatin looping, chromatin accessibility and nearby gene activity affect the chance for an ssDNA site to be selected as the site of crossover formation. First, we noted that the 16,712 ssDNA HSs that are also recombination HSs (ssDNA + /rec_HSs +) were more likely to overlap chromatin loops in early primary spermatocytes compared to the 7986 ssDNA HSs that did not act as recombination HSs (ssDNA + /rec_HSs −) (odds ratio 1.37 (95% confidence intervals [1.32, 1.42])), suggesting a positive association between looping and cross-over formation after double-strand break initiation. Within the set of loop-overlapping ssDNA HSs, associated loop expression levels were *lower* for ssDNA + /rec_HSs + compared to ssDNA + /rec_HSs − (median loop expression levels of 2.6 and 2.1, respectively; Wilcoxon test: *W* = 170,923,365, *p* < 10^ − 16). Similarly, the level of regulatory activity—measured as the number of footprints inside early primary spermatocyte ATAC-seq peaks—was lower at ssDNA + /rec_HSs + compared to ssDNA + /rec_HSs − sites (a median of 2 versus 5 footprints per peak; Wilcoxon test: *W* = 90,436,936, *p* value < 10^−16^), suggesting that gene expression negatively associates with cross over formation at ssDNA sites. Next, we assessed the enrichment of transcription factors motifs at footprinted ssDNA + /rec_HSs + versus ssDNA + /rec_HSs − sites [65, 66]. The PRDM9 (MA1723.1) motif was enriched at ssDNA + /rec_HSs + compared to ssDNA + /rec_HSs − sites (OR = 1.14, log10(*p* value) = 10.24), whereas CTCF (MA0139.1) was enriched among ssDNA + /rec_HSs − sites (OR = 1.35, log10(*p* value) = 15.16). In a ranked list analysis [52] across all DNA-binding proteins that were expressed in early primary spermatocytes, functional GO terms associated with “chromatin insulator sequence binding” and “RNA polymerase II proximal promoter sequence-specific DNA binding” were enriched among ssDNA + /rec_HSs − compared to ssDNA + /rec_HSs + site (Additional file 1: Table S3), again highlighting the negative effect of gene transcription and the presence of insulator sequences on cross over formation. Thus, chromatin looping and gene activity affect downstream processing of double-strand break sites at multiple levels: cross-over formation is favoured *within* loops, but gene activity inside loops counteracts this process, rendering regions with higher regulatory and transcriptional activity less likely to be favoured.

## Discussion

Chromatin loop dynamics during human meiosis are poorly understood and, consequently, the roles of such 3D structures in meiotic cell function are an area of active investigation. Here, we define looping patterns during human spermatogenesis and find dramatic changes between cell types, ranging from expanded loops encompassing much of the genome in early primary spermatocytes (as cytologically described in [5]), to the mature sperm genome, which almost exclusively forms loops near the telomeres In addition, we find that changes in chromatin looping are not strong predictors of gene expression dynamics during meiosis, and no evidence for an enrichment of functional categories of genes inside loops, suggesting that loops largely play an architectural role, primarily facilitating synapsis of homologous chromosomes.

Homologous recombination is invariably linked to the 3D structure of meiotic chromosomes (reviewed in [3]). Here, we use human genomic data to show that early spermatocyte loop structures have an impact on the position of both DNA breakage and cross over events—with DMC1-bound ssDNA sites more likely to result in cross overs if located inside a loop [8]. Further, haplotype block ends are enriched near loop ends, reflecting increased linkage disequilibrium (LD) within prophase I loops—in contrast to results of a previous study that investigated chromatin interactions in somatic cells [67]. However, we note that haplotype blocks tend to be much smaller than loops, on average (see Fig. 6G), and LD will not extend very far in relation to loop size, hence not be sufficient to maintain favourable combinations of promoter and enhancer variants relevant for prophase I. An enrichment of haplotype block ends near anchor points is, however, consistent with our results that recombination often occurs at anchor sites during meiosis, albeit at lower rates than suggested based on ssDNA sites alone. We have shown that chromatin loop anchor sites can be predicted using a computational approach to exploit previously published single cell expression and chromatin accessibility data. Cell states in the spermatogenesis scATAC-seq dataset were identified by stratifying cells based on their accessibility profiles near marker genes, and footprinting analysis was used to computationally identify CTCF-bound sites—based on prior knowledge of characteristic transposase “footprints” near protein-binding motifs [68]. To build even more precise models, it would be desirable to obtain ChIP-seq data of CTCF within individual spermatogenesis cell types in humans, especially since some CTCF-binding sites may be occupied by its meiosis-specific paralogue (BORIS/CTCFL), which does not interact with cohesin [69] but occupies a subset (~ 12%) of CTCF motif sites during spermatogenesis [70]. In contrast to CTCF sites, however, CTCFL/BORIS-binding sites are not oriented in a convergent orientation and are biased towards more accessible chromatin compared to anchor sites [70]—both of which are factors that form part of our model training.

In our study, computationally determined cell states were further corroborated by the strong association of accessible sites and features of recombination that were only found in early primary spermatocytes—a relationship that is only expected if cell populations are accurately determined. Next, we used the information on CTCF-binding, in combination with other features, to identify chromatin loops in meiotic cell populations. In such pools of cells, our training data showed high accuracy in predicting chromatin looping—despite cohesin (RAD21), which facilitates loop extrusion [71, 72]—not being part of the model. This is consistent with reports that features related to CTCF binding may be sufficient to predict looping in most contexts [32]. In addition, looping was accurately predicted in a different cell line with corresponding single cell data.

Meiotic chromatin dynamics are further complicated by the fact that some cohesin subunits are replaced by meiosis-specific molecules (e.g. REC8 instead of RAD21), and the possibility remains that this modification could change the outcome of the loop dynamics that we are aiming to predict. Our model does not take into account large-scale compartment switches and global chromatin changes, which have been observed during mammalian meiosis [20, 21], nor the role of chromosomal axis proteins [73–75] or other factors involved in DSB-initiation, such as the recombination initiation complex (e.g. SPO11), which is known to interact with the chromosomal axis [76, 77]. Hence, it would be worthwhile to extend to more multi-faceted machine learning models that also incorporate larger-scale chromatin changes and information on other players that may drive the positioning of recombination events.

Despite these limitations, our model predicts loop expansion in preparation for the first meiotic division, a phenomenon that has also been observed in murine Hi-C data [28]. However, despite our model allowing for loop sizes of up to 2 Mb, we estimate loop sizes in early primary spermatocytes to have a mean length less than 500 kb, corroborating the notion that computational methods can detect smaller loops that may be overlooked in vivo. Loops are biased towards the tips of the chromosomes already in early primary spermatocytes, where male recombination is known to be enhanced [78]. Later during spermatogenesis, in the compacted sperm genome, loops are largely confined to the tips of the chromosomes. This observation is consistent with experimental data which have shown that telomeric regions are the only regions that retain their histones after the histone-to-protamine transition in haploid sperm; these telomeric ends locate at the nuclear membrane and are free to be actively transcribed in sperm [79]. In sperm, loop sizes in the compacted part of the genome were often larger in size than loops in prophase I; this is also in line with inactive chromosome regions having, on average, larger loop sizes [8, 18]; thus, the phenomenon of enlarged loops in sperm might reflect different processes compared to the expansion of loops in diploid pre-meiotic cells. Overall, our predictions on chromatin looping align with experimental studies [18, 28], while providing the exact positioning of loops in human meiosis—or, rather, a snapshot of loop positions, given that CTCF binds DNA at a much more dynamic way compared to cohesin, with shorter binding times [80], and the process may be more dynamic than captured by our methods.

Another shortcoming of the data analysed here is that looping of the maternal and paternal chromosomes cannot be distinguished—something that could only be achieved using crosses between inbred lines, such as mouse strains. It would also be beneficial to dissect the different stages that make up prophase I, to paint an even higher resolution picture of chromatin dynamics during meiosis. This would require higher numbers of cells in which to estimate loops in our pseudo-bulk analyses. In this study, the PRDM9 genotype of the three South East Asian tissue donors was unknown—and may be a combination of different alleles, given the high level of polymorphism at the PRDM9 locus; thus, we used the combined set of ssDNA hotspots detected across genotypes [54], which in turn limits the accuracy that any random forest model of ssDNA and recombination hotspots can achieve. Finally, the paternal recombination map was derived from the Icelandic population, which is likely to differ between the recombination map in South East Asians, but genetic maps with a similar level of resolution are not available; again, this sets a limit to the accuracy of our modelling. Nevertheless, the positioning of CTCF sites (and hence that of most loops) is likely to be conserved between European and Asians, and the predicted looping structure should be largely transferable between populations.

## Conclusions

Computational predictions of DNA folding are a powerful technique to study the process of meiosis and recombination, in cell populations which are otherwise difficult to study. Our predictions of chromatin loops in human meiosis align with experimental data from other organisms, but the genome-wide resolution obtained by our approach is unprecedented and provides novel insights into the processes that drive human diversity and adaptation.

## Methods

### scRNA-seq processing

Single cell gene expression data of testicular cells were downloaded from the GEO database (Series GSE112013) [34], including the UMI (unique molecular identifier) table for 6490 individual cells. Cluster identity for each cell was downloaded from [34]. Based on marker gene expression, cells had been classified into 13 clusters, including 8 germline cell types at different stages of differentiation as well as 5 somatic cell types [34]. Using Seurat 4.2 in R 4.1.3, we created a Seurat object from these data, using the parameters min.cells = 3, min.features = 200 and followed the standard Seurat pipeline including NormalizeData(with normalization.method = “LogNormalize” and scale.factor = 10,000), FindVariableFeatures(using selection.method = “vst” and nfeatures = 2000), ScaleData() and RunPCA(), finding variable features (), scaling the data and running PCA, using the variable features. After checking the Elbowplot for the number of dimensions to use, we used FindNeighbors(with dims = 1:20) and RunUMAP(with dims = 1:20). We used the original cell type classification [34] to plot the dimension reduction plot using DimPlot(). Aggregate expression values in each cell type were calculated using Seurat’s AggregateExpression(), which also calls ScaleData(). We used GTFtools_0.9.0 to extract the transcription start sites as defined in the gtf file Homo_sapiens.GRCh38.97.chr.gtf, and the scaled expression data were converted to an input expression table as required for the machine learning pipeline (https://github.com/ykai16/Lollipop).

### scATAC-seq processing

Raw sequencing and barcode files were downloaded from the SRA sequence archive (SRR21861961, SRR21861962 and SRR21861963) [13] and processed using 10 × Genomics Cell Ranger’s cellranger-atac count (version 1.1.0). Using standard filtering of low-quality barcodes, this resulted in 20,296 barcodes being assigned to cells. We loaded the filtered peak barcode matrices from the Cell Ranger output into Seurat 4.2 using Read10X_h5() and performed batch correction using Harmony(with reduction = “lsi”), followed by RunUMAP(with dims = 2:30, reduction = “harmony”), and FindNeighbors(with k.param = 20, prune.SNN = 0.3, n.trees = 30).

Cell states were transferred from the scRNA dataset to the scATAC dataset using Signac 1.10.0 [35]. First, we next created a fragment object with CreateFragmentObject() and calculated GeneActivity() in the scATAC-seq dataset using VariableFeatures() of the scRNA-seq dataset. After normalisation and scaling of the gene activity data using default parameters, we calculated “transfer anchors” between the two datasets, using FindTransferAnchors(with reduction = “cca” and k.filter = NA), i.e. including genes of all lengths. We predicted cell states within the scATAC dataset using TransferData() and added this to the meta data of the scATAC dataset using AddMetaData(). Only cells with a prediction score > 0.4 were kept for further analysis.

UMAP plots including the predicted cell identities was visualised using DimPlot(), and, for illustrative purposes, we co-embedded the scRNA and scATAC data on the same plot using merge(), followed by ScaleData(), RunPCA() and RunUMAP().

CallPeaks() was used to call ATAC-seq peaks independently in the thirteen predicted cell types. We created a fragment object of cells whose associated cell-type prediction scores were > 0.4, and then created a feature matrix from which we created a new Seurat object, using CreateChromatinAssay() followed by CreateSeuratObject(), excluding blacklisted regions.

We added motif information and footprinting of CTCF using the Seurat Footprint() function, with “in.peaks = TRUE” and ran RunChromVAR() to visualise the activity of CTCF in the different cell types using FeaturePlot(). Observed CTCF footprints in the different cell types as well as the expected Tn5 insertion enrichment was plotted using the PlotFootprint() function. FindTopFeatures() was used to obtain a measure of peak activity, calculated as the percentile of activity in each cell type separately.

FilterCells() was used to obtain separate bed files of all fragments in the different cell types separately, and fragments overlapping CTCF motif-containing peaks were extracted using bedtools intersect, yielding a file of sequence fragments overlapping CTCF-binding sites.

All output from the initial scRNA-seq and scATAC-se analysis was lifted to the hg19 assembly using liftOver tool.

### GM12878 single cell data processing

We processed single cell data of the GM12878 cell line in a way analogous to the testis datasets.

scATAC-seq data came from the 10X Genomics website, a 1:1 mixture of human GM12878 and mouse EL4 cells (https://www.10xgenomics.com/resources/datasets/1-1-mixture-of-fresh-frozen-human-gm12878-and-mouse-el4-cells-2-standard) with the associated data summary available at https://cf.10xgenomics.com/samples/cell-atac/2.1.0/10k_hgmm_ATACv2_nextgem_Chromium_X/10k_hgmm_ATACv2_nextgem_Chromium_X_web_summary.html. First, we extracted the 4595 cells annotated as “human” (as opposed to “mouse”) and created a chromatin assay, then a Seurat object from those human-derived data. We ran FindTopFeatures() and created a fragment object based on human-only fragments. Using AddMotifs() and Footprint(), we obtained all estimated CTCF-binding sites in GM12878, including, as a measure of peak strength, the percentile of cut sites. A separate bed file contained all sequence fragments overlapping those CTCF peaks.

We investigated the correspondence between our estimated CTCF-binding sites versus experimentally derived CTCF-binding sites in GM12878 by making use of encode ChIP-seq data (accession numbers ENCFF797SDL, ENCFF951PEM, ENCFF796WRU and ENCFF827JRI) and confirmed that the majority of sites were shared between the different datasets (Additional file 2: Fig. S4). Notably, the 12,148 sites that were shared among all 4 ChIP-seq experiments but not found in the scATAC-seq data had lower signal values compared to the 20,066 ChIP-seq-scATAC-seq shared sites (median signal values of 102.4 and 201.5, respectively; Wilcoxon test: *W* = 193,625,454, *p* < 2.2e − 16). Conversely, sites that were only present as CTCF footprinted peaks had a somewhat reduced peak strength compared to sites that were shared with the ChIP-seq data (median percentile cut sites of 0.49 and 0.53, respectively; *W* = 115,733,156, *p* = 1.703e − 09).

Single cell RNA for GM12878 was downloaded from the GEO database (dataset GSM3596321) [81]. We processed the data in the same way as the testis-derived data, including creating a Seurat object from the gene expression matrix (min.cells = 3, min.features = 200), running NormalizeData(), FindVariableFeatures(selection.method = “vst”, nfeatures = 2000), ScaleData() and RunPCA(). We then ran AggregateExpression() to obtain scaled gene expression values for the 18,170 genes provided in the gene expression matrix.

### Adapting a machine learning pipeline to single cell data

To train the random forest classifier [31], we used ChIA-PET (Geo dataset GSM1872886) and Hi-C data (Geo dataset GSE63525) from the GM12878 cell line.

First, we trained the model on 90,211 “true” ChiA-Pet supported loops with a median size of 164,546 bp, using the provided scripts “prepare_training_interactions.py”, “add_features.py” and “train_model.py” (available at https://github.com/ykai16/Lollipop). The parameters of the model were relaxed so as to allow loops of up to 2 Mb, by setting “maxLength” in “prepare_training_interactions.py” to 2,000,000. Positive and negative training interactions were characterised by training features, which included scaled gene expression values, scATAC-seq peaks as well as footprinted CTCF sites and their relative peak strength. Additional features included the CTCF motif score (sequence similarity to the consensus motif, calculated by the original authors using Fimo [82]) as well as the phastCon motif conservation of the CTCF motifs (http://hgdownload.cse.ucsc.edu/goldenpath/hg19/phastCons100way) [83].

We further modified the pipeline to encompass single cell data: the bed file associated with the CTCF peaks contained all fragments overlapping peaks (rather than raw reads overlapping peaks), and, accordingly, we adjusted the “shift” parameter in “add_features.py” to half the median size of the sequence fragments (= 136/2), and also removed the strand information associated with raw reads files. Further, since we have fewer input variables compared to the published pipeline, we changed the parameters “max_depth” and “max_features” in the RandomForestClassifier() function of “train_models.py” to the default values of “None” and “sqrt”, respectively (Scikit-learn package [84]).

The model was evaluated computationally using tenfold cross validation with the “StratifiedKFold” strategy to ensure that each fold had the same proportion of class labels as the original dataset [84]. Cross-validation results, ROC and PR curves were automatically created.

Last, using the script “make_denovo_predictions.py” (also available at https://github.com/ykai16/Lollipop), the trained classifier was used make de novo predictions of chromatin looping in the eight spermatogenesis cell types separately (SSCs, differentiating spermatogonia, early primary spermatocytes, late primary spermatocytes, round spermatids, elongated spermatids, sperm I and sperm II), including the same set of input features as in the model trained on GM12878—again, allowing for interactions of up to 2 Mb by setting “distance_distal = 2,000,000” and adjusting the “shift” parameter in “lib.py” to half the median size of the sequence fragments (136/2).

### Testing the robustness of the machine learning model

We modified the “train_models.py” script further and performed chromosome-based cross-validations instead of random sub-sampling, which can sometimes lead to over-fitting [44]. Using the GroupKFold method from sklearn.model_selection with n_splits = 5, we set group_kfold.split(X, y, groups = chroms) to split the data based on chromosomal location, thereby preventing data leakage from the training into the test sets.

Similarly, we also trained a more simplified model (based on the original re-sampling scheme [31]) by removing gene expression and ATAC-seq peaks from the set of training features—and keeping only CTCF peak strength, conservation and motif orientation as input features. Both alternative models were evaluated using ROC and PR curves.

### Validation of predicted loops in an independent single cell dataset

We tested the performance of the modified random forest classifier on the K562 cell line, using published scATAC-seq [85], scRNA-seq [86] and ChIA-PET high confidence CTCF-mediated loop data [87] (geo datasets GSE162690, GSM5687481, GSM5374829). A Seurat object was created from the scRNA-seq data using the provided barcodes, features and matrix data, and aggregate gene expression values were calculated in the same way as for the GM12878 and spermatogenesis datasets above. Raw fastq files were extracted from the scATAC-seq data, i.e. the “high loading” mixture of ten human cell lines, using Cell Ranger’s bamtofastq, and then processed with cellranger-atac count (version 2.1.0). The 2066 barcodes associated with the K562 cell line [85] were used to subset this dataset, followed by peak calling and CTCF foot-printing analysis in Seurat and Signac. Chromatin loops were predicted in the K562 cell line based on the random forest classifier trained on the single cell GM12878 data, i.e. in a fashion analogous to the spermatogenesis dataset but using K562-specific input parameters of chromatin accessibility (276,933 peaks), CTCF-binding (70,127 active sites) and gene expression. The resulting predicted chromatin loops were compared to “ground truth” ChIA-PET loops of the K562 cell line; first, we calculated the midpoints of each anchor region in the ChIA-PET dataset and defined each experimental loop as extending between these two midpoints. We only included loops of 10 kb to 2 Mb in size, as these limits are also set in the machine learning model. To assess the impact of sequencing depth in the ChIA-PET data, we varied the minimum number of PETs required to call a ChIA-PET loop. Then, we used bedtools intersect to count the number of predicted loops that were also reported experimentally, using a 99% reciprocal overlap criterion.

### Enrichment calculations

Meiotic double-strand break maps (ssDNA maps) in human testes were obtained from [54]. Paternal cross-over maps came from [55]. Based on paternal recombination rate estimates [55], recombination hotspots were defined as regions with a recombination rate ≥ 9.85 centiMorgan per Mb (≥ 10 × the genome-wide average).

To calculate haplotype blocks, we downloaded the phase 3 1000 Genomes SNP and Indel file from http://hgdownload.cse.ucsc.edu/gbdb/hg19/1000Genomes/phase3/. Plink version 1.90b4 was used to filter the vcf files for bi-allelic variants called in the East Asian Ancestry (EAS) population, and haplotype blocks were calculated using the –blocks command with no-pheno-req no-small-max-span –blocks-max-kb 2000. Haplotype blocks of a minimum size of 1 kb were used for further analysis.

The R package genomation 1.26.0 [43] was used to visualise enrichments of sequence features near accessible sites and predicted loops in early primary spermatocytes, including the overlap between loop features and ssDNA overlap, paternal cross-over frequency and haplotype block ends. In the case of accessible sites, we used the resize() function with “width = 6000” and “fix = ‘center’”, followed by the ScoreMatrix() function to calculate genomic enrichments around the accessible sites in all eight cell types. For loops in early primary spermatocytes, we considered the full length of loop coordinates plus/minus 20% of the loop length, and then divided each such region into 140 even-sized bins. The ScoreMatrixBin() function was used to calculate genomic enrichments for each of the 120 bins—which varied in absolute size among loops.

The R package regioneR 1.26.1 [88] was used to perform circular permutations of sets of genomic regions, i.e. early primary spermatocyte loop anchor points or loops, respectively, versus haplotype blocks, ssDNA peaks and paternal cross over events. We used the permTest() function with parameters ntimes = 1000, randomize.function = circularRandomizeRegions, evaluate.function = numOverlaps, genome = hg38_masked, alternative = “auto”, where hg19_masked = getBSgenome(“BSgenome.Hsapiens.UCSC.hg19.masked”). This yielded a genome-wide estimate of enrichment of overlap between features as measured by *Z*-scores as well as the ratio of observed to expected overlaps.

### Motif analysis

To facilitate de novo motif discovery, the “rgt-hint footprinting” algorithm was applied to scATAC-seq peaks in early primary spermatocytes and their corresponding bam file of aligned reads. The thus obtained 481,840 footprints were intersected with ssDNA peaks [54], and FASTA sequences of the 93,681 footprints inside ssDNA peaks were subjected to the MEME de novo motif search algorithm [58], using the parameters -nmotifs 10 -minw 6 -maxw 50. Motifs were passed onto the Tomtom algorithm [89], using the default parameters -min-overlap 5 -mi 1 -dist pearson -evalue -thresh 10.0, thus searching for similar, previously described, motifs among the de novo motifs inside ssDNA HSs.

Footprints inside ssDNA peaks were further processed using the JASPAR enrichment tool (https://jaspar.elixir.no/enrichment/) [65, 66], searching against a known database of DNA binding motifs, i.e. all available human JASPAR 2022 TFBS sets (available at https://zenodo.org/records/6860555) with detectable expression levels in early primary spermatocytes. Footprints were divided into those that overlapped either ssDNA + /rec_HSs − or ssDNA + /rec_HSs + sites. Next, we used the JASPAR_enrich.sh script with the “twoSets” parameter, testing for differential TFBS enrichment between the two sets of genomic regions. Motifs were ranked by their odds ratio of enrichment in ssDNA + /rec_HSs + versus ssDNA + /rec_HSs − and vice versa, and the ranked lists of corresponding transcription factors were compared using the GOrilla tool at https://cbl-gorilla.cs.technion.ac.il [52].

### Calculation of telomere distances

To calculate the distance of a genomic feature (CTCF motif of loops) to the nearest telomere, we calculated the distance of the 5′ and 3′ end of the feature to the 5′ and 3′ end of the chromosome and selected the smallest of these four distances. To calculate the *relative* distance to the nearest telomere with respect to chromosome length, we simply divided telomere distance by chromosome length. Thus, if features are randomly distributed, we expect, on average, a relative telomeric distance of 0.25.

### GO analysis with respect to looping features

GO analysis was carried out using the GOrilla tool at https://cbl-gorilla.cs.technion.ac.il [52]. For all eight germline cell types, we extracted all genes whose promoter sequence overlapped a predicted chromatin loop in the given cell type and ranked these genes by their scaled gene expression values. A second list of genes included all genes whose promoter sequence did *not* overlap a predicted chromatin loop in a given tissue, and this list was also ranked by gene expression levels. Next, we ran the GOrilla tool on each ranked list of genes.

### Gene expression and looping changes

We examined if changes chromatin loops in early primary spermatocytes were associated with gene expression changes by considering cell types with “private” loops, i.e. loops that were predicted to be present only in a single cell type; this included 2166 loops in early primary spermatocytes, 165 loops in late primary spermatocytes, 69 in differentiating spermatogonia and 53 in sperm II (Fig. 4).

We applied Seurat’s FindMarker() function to calculate gene expression changes (log2 fold-change of expression) in the comparison of early primary spermatocytes versus each of the other three cell types, using the parameters “min.diff.pct = 0, logfc.threshold = 0, min.pct = 0.1, min.cells.group = 3”. Next, we intersected gene promoters (defined as the transcription start site plus/minus 2 kb) with loops that were found (a) exclusively in early primary spermatocytes and (b) exclusively in differentiating spermatogonia, late primary spermatocytes or sperm II, respectively. The median log2 fold-change of expression was compared between the two categories of genes—promoters in early primary spermatocyte-specific loops or promoters in the respective other cell type’s loops—using a Wilcoxon sign rank test.

### Random forest model

Random forests for classification and regression were implemented using the scikit-learn python package [84]. First, the hg19 genome was subdivided into 2,599,489 1-kb windows, excluding encode blacklisted regions as well as windows containing “NA” values for any of the predictor variables. For each window, the following sequence features were calculated (using bedtools map, betools intersect and custom R and python scripts, respectively): the average GC content; the distance to the nearest telomere; the average meiotic replication time derived from meiotic S-phase nuclei (DNA content: 2-4C; SYCP3-positive and DMRT1-negative) [63]; the number of genes overlapping the window (based on the ensemble annotation “Homo_sapiens.GRCh37.82.chr.gff3”); the number of predicted loops and loop anchor points overlapping the genomic window; the maximum strength of CTCF-anchors in the interval; scaled gene expression values of genes and the average scATAC-seq signal within a window. Cell-type specific features (ATAC-seq signal, gene expression values and looping features) were specific to early primary spermatocytes. Loop anchors were defined as 5 kb blocks of sequence surrounding the anchor CTCF site, and loop anchors occurring “internal” of other loops (i.e. overlapping larger loops) were excised from the genomic interval of a loop, in order to avoid the presence of anchors within loops; loops were pruned by 2.5 kb on either end, to remove upstream and downstream anchors. Spearman correlation coefficients between numerical variables of the model were visualised using the seaborn package [90].

In the random forest classifier model of “ssDNA hotspot overlap”, we modelled the presence of double-strand break hotspots from [54] and included all hotspots observed in any of the AA1, AA2, AB1, AB2 and AC individuals (with the genotype referring to the PRDM9 allele of the respective donor).

In the random forest classifier model of “paternal recombination hotspot overlap”, we modelled the presence of hotspots defined as genomic intervals for which the paternal recombination rate was ≥ 9.85 cM/Mb [55].

In both models, the input data were subdivided into training and test sets (70% of data in the training set) and best model hyperparameters were searched using the RandomizedSearchCV() function of the scikit-learn package, sampling 10 hyperparameter values of “n_estimators” (values between 50 and 200) and “max_depth” ( values between 1 and 20), with fivefold cross-validation. Following this procedure, we built final “best” models with 111 trees and a maximum depth of 18 for ssDNA hotspots, 62 trees with a maximum depth of 16 for recombination hotspots and 158 trees with a maximum depth of 19 for haplotype blocks.

## Supplementary Information

Additional file 1: Table S1 Confidence of loop predictions using ChIA-PET as a control. Table S2 GO enrichments in eight germline cell types. Table S3 Functional GO enrichments at ssDNA sites that do not progress as recombination hotspots (ssDNA + /rec_HS − compared to ssDNA + /rec_HS +).

Additional file 2: Fig. S1 A comparison of machine learning models to predict CTCF loops. Fig. S2 DNA accessibility signal around germ cell marker genes. Fig. S3 Motif matches within footprints of early primary spermatocyte peaks. Fig. S4 Comparison of CTCF ChIP-seq peaks versus footprinted scATAC-seq peaks in GM12878.

Additional file 3: Genomic coordinates (hg19) of predicted chromatin loops in the eight germline cell types.

Additional file 4: Table S4 Datasets used in this study.

## Data Availability

All data used in this study are publicly available; relevant accession numbers can be found in Additional File 4: Table S4.
