# Supplementary material for: CTCF-anchored chromatin loop dynamics during human meiosis
Source: BMC Biol. 2025 Mar 20;23:83. doi: 10.1186/s12915-025-02181-3 (PMC11927364; doi:10.1186/s12915-025-02181-3)
Supplement: Supplementary file 2 — Additional file 2: Fig. S1 A comparison of machine learning models to predict CTCF loops. Fig. S2 DNA accessibility signal around germ cell marker genes. Fig. S3 Motif matches within footprints of early primary spermatocyte peaks. Fig. S4 Comparison of CTCF ChIP-seq peaks versus footprinted scATAC-seq peaks in GM12878. [file 12915_2025_2181_MOESM2_ESM.docx]

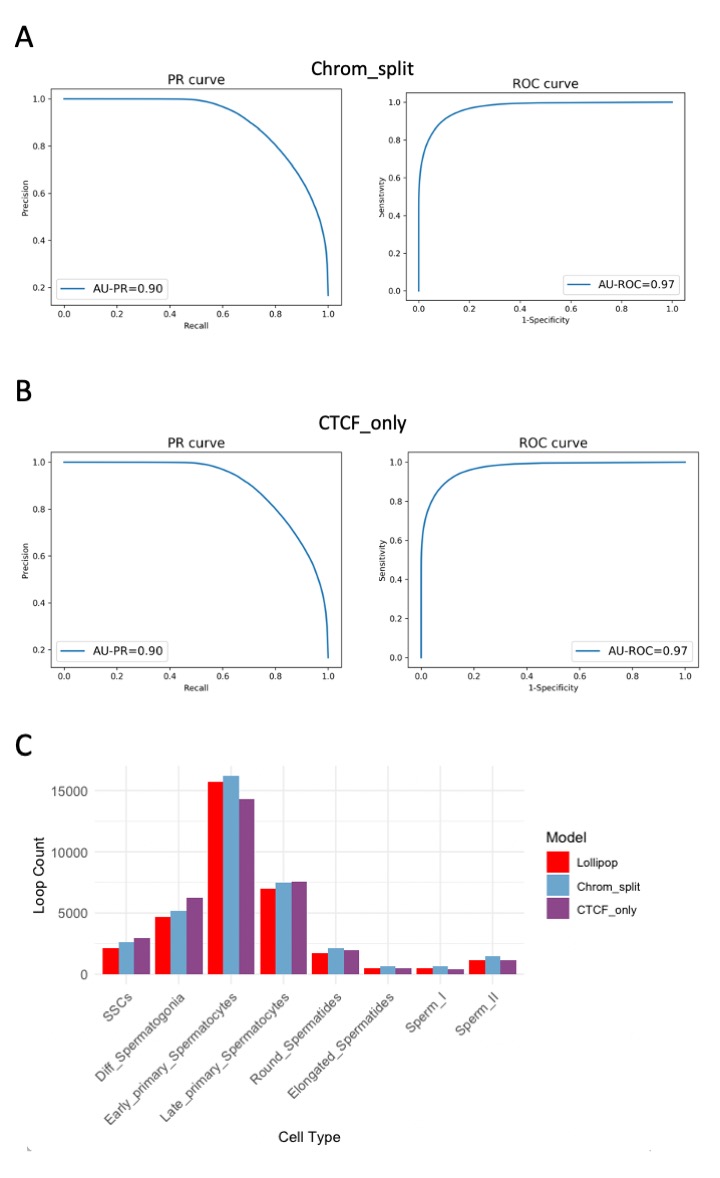


**Supplementary Figure S1: A comparison of machine learning models to predict CTCF loops.**

Model performances (PR and ROC curves) of models trained (A) with chromosomal training/test splits – the “Chrom_split model” - and (B) a reduced model that only uses CTCF-related features as training features – the “CTCF_only model”. (C) Comparing predictions of the number of CTCF-anchored loops based on (A) and (B) to those of the main model used in the manuscript (the “Lollipop” model).


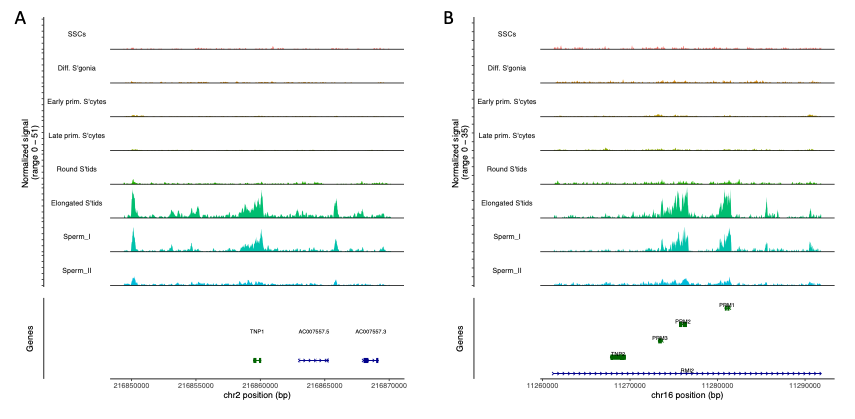


**Supplementary Figure S2: DNA accessibility signal around germ cell marker genes.** Accessibility signals are shown for each of the eight germline cell types separately, along the sequence su­­­rrounding the TNP1 (A) and PRM2 (B) gene. Both genes are preferentially expressed post-meiotically (34) and involved in the histone to protamine replacement during the haploid phase of spermatogenesis.


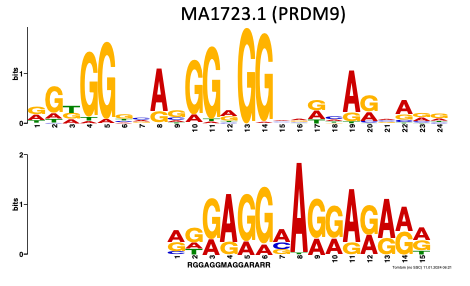


**Supplementary Figure S3:** **Motif matches within footprints of early primary spermatocyte peaks.** The PRDM9 motif in the JASPAR 2022 CORE database (top) and its matching motif inside footprints that also overlap ssDNA hotspots (bottom). Tomtom similarity q-value = 0.019, Benjamini-Hochberg corrected.

**Supplementary Figure S4: Comparison of CTCF ChIP-seq peaks *versus* footprinted scATAC-seq peaks in GM12878. (**A) An IGV browser screenshot shows the locations of GM12878 ChIP-seq peaks as well as scATAC-seq peaks containing a footprinted CTCF motif. (B) An upset plot of the same data shows the intersection sizes between the datasets. The encode ChIP-seq datasets used in this comparison include datasets ENCFF797SDL (ChIP-seq_1), ENCFF951PEM (ChIP-seq_2), ENCFF796WRU (ChIP-seq_3) and ENCFF827JRI (ChIP-seq_4).
